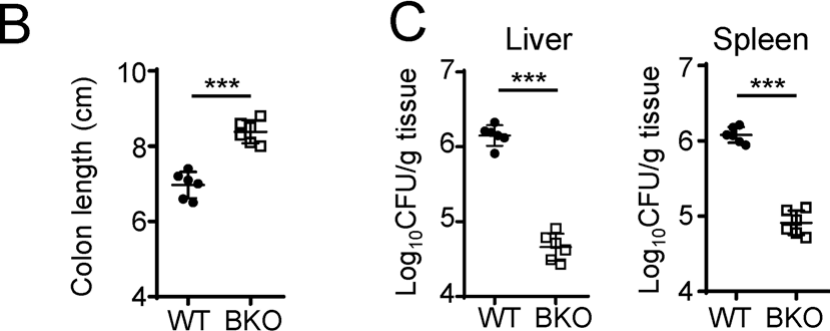# Correction: TRAF3–EWSR1 signaling axis acts as a checkpoint on germinal center responses

**DOI:** 10.1084/jem.2022148307122023c

**Published:** 2023-07-18

**Authors:** Yanchuan Li, Lele Zhu, Chun-Jung Ko, Jin-Young Yang, Hongjiao Wang, Ganiraju Manyam, Jing Wang, Xuhong Cheng, Shuli Zhao, Zuliang Jie

Vol. 220, No. 8 | https://doi.org/10.1084/jem.20221483 | April 25, 2023

The authors regret that in the originally published article, the plots in Fig. 7 B (depicting the colon length of WT and *Ewsr1*^BKO^ mice) and the left panel of Fig. 7 C (depicting the bacterial load in the liver of WT and *Ewsr1*^BKO^ mice) were inadvertently switched during the revision process. The corrected Fig. 7 (B and C) is shown here. This correction does not change the original conclusions of the manuscript, and the legend remains unchanged. The error appears in PDFs downloaded before July 12, 2023.

**Figure:**